# What is a research derived actionable tool, and what factors should be considered in their development? A Delphi study

**DOI:** 10.1186/s12913-018-3551-6

**Published:** 2018-09-27

**Authors:** Susan Hampshaw, Jo Cooke, Laurie Mott

**Affiliations:** 1 School of Health Related Research, The University of Sheffield, Sheffield, UK; 2 Health and Social Care Research, Collaborations for Leadership in Applied Health Research and Care Yorkshire and Humber (CLAHRC YH), Sheffield Hallam University, Sheffield, UK; 3 Doncaster Metropolitan Borough Council, Doncaster, UK

**Keywords:** Delphi study, Knowledge transfer, Dissemination, Knowledge translation, Research derived actionable tool (RDAT)

## Abstract

**Background:**

Research findings should be disseminated appropriately to generate maximum impact. The development of research derived ‘actionable’ tools (RDAT) as research outputs may contribute to impact in health services and health systems research. However there is little agreement on what is meant by actionable tool or what can make them useful. We set out to develop a consensus definition of what is meant by a RDAT and to identify characteristics of a RDAT that would support its use across the research-practice boundary.

**Methods:**

A modified Delphi method was used with a panel of 33 experts comprising of researchers, research funders, policy makers and practitioners. Three rounds were administered including an initial workshop, followed by two online surveys comprising of Likert scales supplemented with open-ended questions. Consensus was defined at 75% agreement.

**Results:**

Consensus was reached for the definition and characteristics of RDATs, and on considerations that might maximize their use. The panel also agreed how RDATs could become integral to primary research methods, conduct and reporting. A typology of RDATs did not reach consensus.

**Conclusions:**

A group of experts agreed a definition and characteristics of RDATs that are complementary to peer reviewed publications. The importance of end users shaping such tools was seen as of paramount importance. The findings have implications for research funders to resource such outputs in funding calls. The research community might consider developing and applying skills to coproduce RDATs with end users as part of the research process. Further research is needed on tracking the impact of RDATs, and defining a typology with a range of end-users.

**Electronic supplementary material:**

The online version of this article (10.1186/s12913-018-3551-6) contains supplementary material, which is available to authorized users.

## Background

Considerable resources are invested in applied health and social care research yet there is an acknowledged gap between what is known and what is acted upon [1]. There is a growing interest in addressing this gap, and a simple starting point is the need to communicate research findings appropriately to audiences that are in a position to act. Research funding bodies expect their grant holders to disseminate their findings however, there is a lack of clarity about what is meant by dissemination [2]. Wilson et al. in their scoping review of conceptual frameworks to support dissemination identified 33 frameworks. Only 20 of these had theoretical underpinnings, for example on the use of persuasive communication such as within the Lavis framework [2, 3]. In addition, few funding bodies offer guidance on dissemination beyond the need to do so [2].

We acknowledge that there is considerable interest in co-produced approaches to research planning, activity, and dissemination, and there are a number of initiatives funded with this in mind. Examples include the Integrated Knowledge Translation Research Network in Canada, and the National Institute for Health Research Collaborations for Leadership in Applied Health Research and Care (NIHR CLAHRC) in England. These collaborations are designed to better bridge the knowledge to action gap [4–9].

However, it is not unreasonable to suggest that the default dissemination approach for researchers’ is concerned with the conduct and reporting of research in a high quality ‘peer reviewed’ journals. System factors drive these behaviours, for example, developing an academic career requires evidence of a publication track record which despite the rise of altmetrics [10] is still largely viewed through the lens of scientific journals and their associated impact factors. In the UK, this is reinforced by metrics related to the Research Excellence Framework (REF) where institutions (and arguably careers) are measured against publications in high quality, international journals. The introduction of case studies demonstrating societal impact may ameliorate this, and Greenhalgh and Fahy’s review of the 2014 submissions identified targeted knowledge translation activities in 38 of the 162 impact case studies [11].

Academic publications serve the scientific process but have limitations in terms of bridging the gap between knowledge and action [12]. Given, as we outline above, that many of the quality and esteem criteria in academia are related to such publications, we acknowledge that they should be part of the dissemination process, and they are also an important step in the production of summary evidence on what is known. Graham’s conceptualisation of knowledge into action, for example, divides the process into two concepts: knowledge creation and an action cycle [1]. He suggests that as well as reporting in peer reviewed journals, researchers have an additional ethical duty to publish research results which are capable of ready implementation, and which may support the knowledge into action gap. He uses a funnel to symbolise knowledge creation and argues that as (empirically derived or experiential) knowledge travels through the funnel it is increasingly tailored to meet the needs of stakeholders. At the end of the funnel the ‘third generation’ knowledge takes the form of ‘tools’ or products for example, guidelines or decision aids. These tools are designed to present the knowledge in a format that meets stakeholders’ (end users’) needs [1]. This is a helpful model which conceptualises two elements to knowledge mobilisation and their connectivity.

Our study focuses on the knowledge creation element of this cycle, and seeks to identify further methods of disseminating knowledge beyond conventional scientific publication in health services and health systems research. Our contribution to this field stems from experience within one of the NIHR CLAHRCs in the UK. These are collaborations tasked with addressing the research-practice gap through partnership between academia and practice. Many CLAHRCs operate through co-producing knowledge within these partnerships, which in itself supports research mobilisation into practice. Rycroft- Malone et al.’s evaluation of the CLAHRCs suggests that their potential *‘to close the metaphorical ‘know–do’ gap was dependent on historical regional relationships, their approach to engaging different communities, their architectures, what priorities were set and how, and providing additional resources for implementation, including investment in roles and activities to bridge and broker boundaries’* [8].

Working within a CLAHRC has afforded the opportunity to reflect (using our case study library available from http://clahrc-yh.nihr.ac.uk/resources/case_study_library to identify research outputs that may have potential in crossing the boundary into policy and practice. For example, some of the outputs from the Keeping Warm in Later Life (KWILLT) project [13] went beyond academic publication and used a segmentation model to produce 6 pen portraits to summarise the research findings for policy makers. Pen portraits are descriptive narratives to explain health behaviours in complex environments. In the KWILLT project the pen portraits described groups of older people who are vulnerable in cold weather, and the reasons that make them vulnerable. These were developed in order to inform targeted interventions for these groups in policy and practice. Specifically, the pen portraits helped both to make the research visible and facilitated action on the part of local and national commissioners. [14] The pen portraits were used as a resource in the National Cold Weather Plan; they informed policy making in local government, and supported volunteer organisations to target advice to vulnerable older people. As Rycroft- Malone et al. [4] acknowledge, some effort is needed to plan cross boundary work and the CLAHRC pen portraits supports this. We think the RDATs are a promising option as they can act as a ‘boundary object’ [4, 15] which Fox [15] describes as a construct to improve the uptake and transfer of research into other areas such as social policy and public services. RDATs then could represent ‘tailored’ knowledge [1] which is potentially immediately useful to policy and practice. This idea, albeit requiring testing and refining, seems to be potentially helpful.

The term ‘actionable is repeatedly used in the implementation science literature occurring, for example, 843 times within the journal *Implementation Science*. It is often the basis of discussion in both old and new media presentation of scientific findings, but there is little conceptual clarity around this term. We suggest it is generally understood in quite broad terms, and taken as meaning that the findings of a research study are implementable by practitioners or policy makers, and is useful to them. Further, in applied research settings it is arguably a given that there is a need for research to be actionable (directly) or inform action (conceptually). However, there is likely less clarity on what constitutes an actionable output and little evidence on how to produce RDATs nor any debate on where such tools fit in evidence hierarchies [16].

Our study sets out to address this first issue, and aims to contribute to understandings about how to develop a RDAT, and in particular the importance of resource, end user involvement and embedding of such activity in the research process. The focus of this study was to seek some clarity about research outputs that might help to translate research for policy makers and practitioners. In the context of this study the end user are therefore policy makers and practitioners. Although we have consulted patient representatives on some decision making (see methods section), they are not the intended target audience for policy and practice related RDAT, so are therefore out of scope. Our aims were firstly, to develop a consensus definition of what is meant by the term ‘actionable tool’. Secondly, to come to some consensus on characteristics of an actionable tool that would support its use across the research-practice boundary, including how they might be integrated into the methods, conduct and reporting in projects. We also hoped to gain some agreement of examples of RDAT. Finally, throughout the study process we have reflected on the emerging definition and its potential to contribute to crossing the know-how boundary.

## Methods

### Study design

We chose a modified Delphi technique which is an ‘iterative multistage process designed to combine opinion into group consensus [17].’ It is a widely used method in health care aimed at enhancing decision making [18] which seeks opinion from a board range of experts [17, 19]. The main uses of Delphi technique in healthcare includes priority setting and gaining consensus on an issue where none previously existed [18, 20], such as the definition and characteristics of a RDAT.

The Delphi technique is usually done through a series or ‘rounds’ of questionnaires, where each independent respondent is able to use their expertise to consider, in this case a definition and assess its utility using a series of Likert-type scales [21]. The key features of the method are anonymity between participants, with structured feedback on the panel’s level of agreement to each question [18]. Panel members are then invited to complete another questionnaire with a view to reaching agreement.

### Participant recruitment and inclusion criteria

We elected to undertake a modified Delphi; the stages are set out in Table 1.The Delphi approach is particularly useful to engage ‘informed individuals’ across multiple locations and from a diverse professional and other relevant backgrounds [22]. Diamond et al. [23] advise that it is important to qualify how the panel is selected for the study quality. For the purpose of this study key stakeholders included professionals, policy makers and researchers on both sides of the research-practice gap, and those undertaking research and implementation projects in knowledge mobilisation and knowledge transfer linked to the CLAHRC. All stakeholders belonged to an organisation or research/ implementation group that had responsibility to promote the use of research in practice. Participants were purposively selected by stakeholder groups.(see table one for more detail). These experts were approached by an email including a brief outline of the project, its aims, expected number of rounds, and anticipated time commitment. This resulted in a panel of 33 experts, comprising of researchers, research funders, policy makers and practitioners Although 10 participants did not complete the 3rd round, we were able to maintain membership sufficient to produce consensus [18].

**Table 1:** Detail of participants in each round

Round	Purpose of round	Participants
1. Workshop activities (*n* = 10)	Developing concepts based on expert opinion and insights from knowledge translation literature (personal libraries of members and facilitator) and to develop the initial definition	Knowledge mobilisation experts in CLAHRC Yorkshire and Humber Knowledge into Action Theme. Some of whom are considered international experts.
2 Online survey (*n* = 33)	To develop consensus against the initial definition and to test example RDAT against this definition.	*Experts with an interest in knowledge mobilisation*. These were invited from members of two knowledge mobilisation themes in a CLAHRC in the north of England. They included both academics and clinicians from a range of backgrounds including doctors, nurses, Allied Health Professionals and National Health Services (NHS) managers and commissioners. They also included members from an Academic Health Science Network, and NIHR Knowledge Mobilisations Fellows. Academics within this group included representation from the disciplines of information science, design, sociology and psychology. All were involved in the development or research projects in knowledge mobilisation or research use in practice. All of these experts are UK based, but some have international profiles. *International experts in health services research*. This included academics who had an international expertise in this area of health services and health systems research, and research in implementation science. All were international experts present on at least one CLAHRC advisory panel, and one was based outside the UK. *Commissioners of health services*. The NHS is divided into provider and commissioners of services. This stakeholder group included participants from Clinical Commissioning Groups and a national commissioning organisation called NHS England. *Practitioners and managers in NHS organisations*. These were invited from a Community of Practice aimed at improving research capacity development in NHS organisations. They included representatives from teaching hospitals, smaller district hospitals, community and mental health service providers in the north on England and Scotland *National organisations with a responsibility for dissemination of research findings*. This included participants from the National Institute for Health and Care Excellence (NICE) and the NIHR centre dissemination
PPI Consultation workshop	Mid point findings and helped to inform the next stage of data collection. From round 2 with the PPI group	PPI group from within the CLAHRC
3 - Online survey (*n* = 23)	Feedback on level of consensus. Agreement on the refined definition and new items drawn from analysis of the qualitative responses from round 2	Participants from round 2

In addition to the panel we consulted with a public and patient advisory group between rounds two and three. This consultation helped us sense check mid point findings and informed the content of round 3, specifically that RDAT should be defined by the targeted end user. This will be discussed in more detail in the findings section of the paper.

### Ethical considerations

The Delphi panel members were informed in the invitation email and accompanying material that they were free to withdraw at any point. Experts agreed to be included via email and on-going implicit consent was assumed upon completion of each survey round. The research ethics committee of Sheffield Hallam University approved the study.

### Data collection

Three rounds were administered including an initial workshop, followed by two online surveys. Participants received an email link to the survey and one reminder email. The surveys themselves are available as supplementary files.

### Round 1: Workshop

We adopted an approach advocated in the literature which describes modified Delphi as having a first round that includes face to face contact with experts [19]. We held a workshop that lasted 3 hours with 10 knowledge mobilisation experts from within the CLARHC, some of whom were clinical academics. The workshop utilised a series of activities to explore the concept and definition of a RDAT, to identify the typology of such products, and define their components. The workshop had at its starting point two theoretical models in the knowledge mobilisation literature: boundary object theory used frequently in design [15], and the Graham funnel model [1] within the knowledge into action cycle. Both models are used in the CLAHRC themes involved in the workshop. The discussion points were written up and a working definition was developed which we shared with the workshop members for comment. Members of the panel also signposted us to relevant literature and/or examples of possible actionable tools at this point.

### Round 2: Online survey

**Fig. 1:**
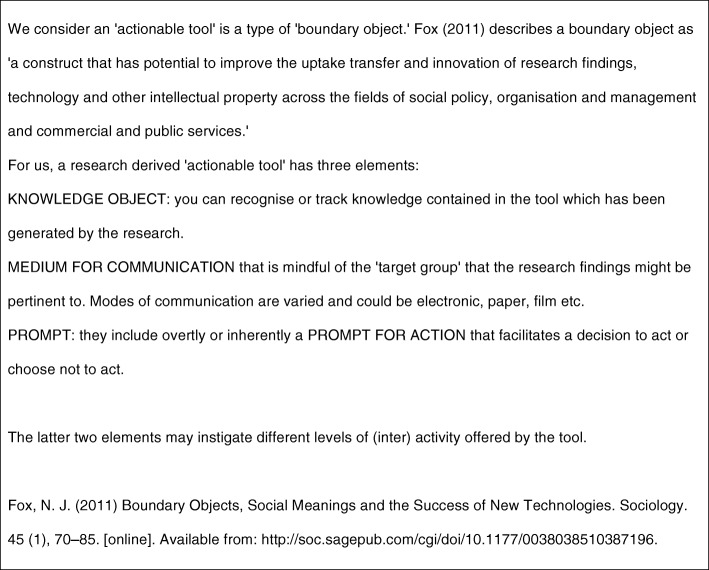
First definition of an actionable tool developed from round one discussion for wider panel to consider in round 2

The information collected in round 1 informed the content of the online survey (see Additional file 1 Round2) which was sent out to the wider panel of experts. In this round experts were asked to comment on the extent to which they agreed with a definition of ‘actionable tool’ (see Fig. 1). It is important to note that we only offered one definition for comment and that this had been developed via the workshop. We also gave the panel examples of items that could be considered to be an actionable tool. A list of such tools were initiated in the first round workshop, and a literature search highlighted some other candidate RDATs. The experts were asked the extent to which they agreed that each candidate item was a RDAT. At all points in the Delphi study participants were able to explain their reasoning using open text boxes within the survey. Finally, participants were asked questions on the necessary requirements within the research process itself to support the production of RDATs.

### Round 3: Online survey

**Fig. 2:**
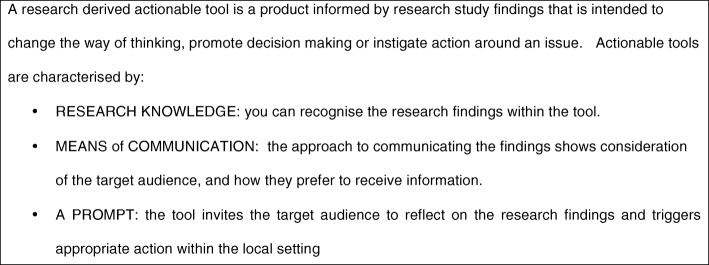
Definition of research derived actionable tool developed from the 2nd round responses, agreed by the expert panel in round 3

Some questions in the survey changed between rounds due to consensus being reached in some areas. Panel members received feedback on the aggregated scores and a summary of the qualitative answers (see Additional file 2 Round3). We also modified the definition based on feedback from respondents, and sought consensus on this adapted definition (see Fig. 2). After analysis and discussion with Patient and Public Involvement (PPI) representatives we elected to remove any further questions relating to the examples of RDAT as we did not gain consensus, and were unlikely to do so. Our PPI advisory group, and second round qualitative responses suggested that in order to make an informed decision about a whether a particular research output was ‘actionable’, more detail was needed about the tool itself, the context of its use, and whether the target user perceived it to be useful in promoting action. The need for establishing views of end users in this respect therefore is important. As a consequence of this finding we concluded that a consensus of panel experts was an inappropriate way to determine this, and did not pursue this broader aim further in round 3 Instead, further questions were prioritised to explore considerations in the shaping the tool’s structure, content and dissemination, all of which were shaped by the round 2 qualitative responses.

### Data analysis

Outputs of the workshops included audio recording, visible records (photographs of completed flipcharts) and summary documentation which was shared with participants (see supplementary file). These were reviewed and used to develop the content of round 2. In rounds 2 and 3 respondents we invited to state their level of agreement to each statement on a five point Likert scale where 1 = strongly agree, 5 = strongly disagree. The panel’s responses to the questionnaire were analysed by calculating group percentages for each item. Consensus was defined at 75% of agreement in the group. This is where the aggregated categories of strongly agree and agree together reached a level of 75% and over, with a median of 1–2. If this level of consensus was attained then the item did not go through to the following round, and consensus reported back to the panel. Items were dropped if the panel reach a consensus disagreement, where strongly disagree or disagree was 75% or over and where the median of 4–5. The study aimed to only run two questionnaire rounds, in order not to over burden the panel and to avoid survey fatigue [19]. If items did not reach consensus, then they are not reported here.

Respondents were able to add free text under each item to further elucidate their reasoning and these were thematically coded and fed back to the group as a commentary. Additionally, some issues were used to develop further Likert questions for consideration in round 3.

## Results

### Results of the first round

The key product of round one was the initial actionable tool definition (see Fig. 1). Participants also identified some factors in the research process that might be considered important in the development of appropriate actionable outputs from research, including the use of co-productive techniques to support this. Such issues were explored in round two.

The workshop also identified some examples of research outputs that might be considered a RDAT such as patient decision aid, clinical protocol, algorithm, and balanced score card. This initial list was used to structure items in the Delphi which asked whether an example could, using the definition, be considered to be a RDAT.

### Results of the second round

#### Definition and characteristics of RDAT

In this round we gained consensus in a number of areas. We did not get agreement on first candidate definition of an actionable tool (Fig. 1) as the definition was considered too abstract and academic by our panel. However, we did get consensus that a definition of RDAT should include distinct descriptive elements (79.41% agreement, median 2). We also had some consensus in what these elements might include. These were that it should include the medium for communication mindful of the target audience (76.47% agreement, median 1) and that it should have a prompt (76.47% agreement, median 2), although the qualitative data revealed additional complexity associated with a prompt. Consensus was nearly reached on the ‘knowledge object’ characteristic of the tool (73.5% agreement, median 1.5), whereby the research knowledge is contained, and can be identified in the tool.

We found stronger, and more consensus around factors that would helpful in the development of actionable tool within the research process including the notions of involvement of end users from early stages, including at the grant application (80.65% agreement, median 1), and the research design (83.37% agreement, median 1). It was also seen as important that funds and resources should be allocated in the project budget (80.65% agreement, median 1). The expert panels’ responses around the definition enabled us to develop a second adapted definition (see Fig. 2), and thematic analyses of their open text answers suggested the other considerations, which contributed to the round 3 questionnaire.

#### Examples of RDATs

We asked the panel’s opinion on whether the list of research outputs identified in round one could be examples of RDAT. Panel members were given an example to help with their decision making. Consensus was only nearly reached for one example, the patient decision aid (75.8%; median = 2) and none of the examples was completely rejected by the panel. Table 2 below illustrates the diversity of views in relation to our other example RDATs.

**Table 2:** Delphi panel scores for example RDATs

Extent to which the listed ITEM is agreed to be an actionable tool (reproduced in the order they appear in the survey)	Strongly agree, agree (%)	Strongly disagree, disagree (%)
Service specification	51.5	15.2
Service evaluation/research tool	48.5	21.3
Worksheet	39.4	27.3
Simulation model	21.2	24.3
Clinical decision aid	57.6	18.2
Audit Tool	39.4	36.4
Executive summary	27.3	57.5
Patient decision aid	**75.8**	12.1
Algorithm for clinical decision making	66.6	15.2
Risk Assessment Tool	51.5	21.3
Balanced Scorecard	24.3	21.3
Teaching and learning pack	45.5	15.2
Local protocol	57.6	24.2
National protocol or guideline	48.5	21.3
Social marketing materials	24.3	39.4
Film	15.3	42.4
Patient Reported Outcome Measure	30.4	27.3
Patient Reported Experience Measure	27.3	30.4

Entry in bold signifies panel consensus

Panel members were able to offer explanation of their scoring. In terms of assessing whether a potential tool was a RDAT, the main difficulty was the lack of consensus (as yet) around a definition. A key message from the panel’s comments were around understanding the context in which the tool could be used, exploring support needed in using the tool, as well as the views of the end user in how useful it might be in a given context. This was further reinforced through discussing these early findings with our PPI advisory group. We also explored the types of tools identified in round one with PPI advisory group, and gave an example of the patient decision aid, illustrating this with a real example of a cataract patient decision aid. The tools were viewed quite negatively by the PPI group who valued the ‘expertise’ of discussions with their clinician rather than the interactive tool. They also felt the tools made assumptions about what was important to them in terms of decision making.

### Results of round 3

We experienced 30% drop out rate of participants between rounds two and three. Twenty-three responded to the survey of the 33 invited. However, we achieve consensus on the new definition given in Fig. 2 (78.2% agreement, median 2).

The new definition was well received. It was viewed as clear and concise and *‘helps the user consider the important elements for translating research findings into everyday practice’ (round 3 participant).* The written responses identified further nuances and understandings, particularly in relation to the prompt and its potential for action. Participants continued to identify the importance of context and the impact of external factors in terms of whether something was implemented or not. For example, the tool might aim to prompt behaviour but external factors to the setting might be the instrument of action, for example, a policy directive or a budget necessity. Another clear view was that the prompt within the tool could aim to encourage discussion within a team or organisation that may or may not lead to action. Although consensus was reached on the visibility of the research within the tool, there was debate around the importance of this to the end user, linked to their knowledge beliefs in deciding whether to use it, or whether it would promote action.

Higher levels of agreement were achieved in the generic content enabling factors that might make the tool more used and therefore promote action. These findings are reported in Table 3. Two of the questions related to what to communicate about the research. Qualitative responses highlighted that understanding the context of the research and its distinctive message was thought to enable the end user to assess its transferability to their context, and identify its unique worth: *‘what is this telling me and next- is this relevant to [my] work area?.’* It was thought that a clear articulation of these two areas is likely to lead to behaviour change.

**Table 3:** Summary results for 3rd round of the Delphi

Question (to what extent do you agree that the following considerations are important to end users of an actionable tool?)	Consensus > or equal to 75% (agreement in %)	Median	Inter-quartile range
Ability to tailor to local context	95.7	1	0
Information on outcome measures to support the tool in practice	95.6	2	1
Study setting or context	91.3	2	1
Information on who needs to take action	91.3	1	0
Information about what the study adds to the evidence	87.0	1	1
Information to support implementation	82.6	2	1
Testimonies from other users	82.6	2	1
Inclusion of evaluation tools	82.6	2	1
The tool prioritises actions	78.2	1	1
Timescales for implementation	78.2	2	1

In the second set of questions, consensus was achieved in all items with the exception of the tool including self assessment guidance for its use (73.9% agreement). There was clear consensus that end users would find the following items important: information on who needs to take action (91.3% agreement); ability to tailor to local context (95.7% agreement); information on outcome measures to support the tool in practice (95.6% agreement) information to support implementation (82.6% agreement); testimonies from other users (82.6% agreement); inclusion of evaluation tools (82.6% agreement); the tool prioritises actions (78.2% agreement); and details timescales for implementation (78.2% agreement). This suggests our experts had clear views about what a tool should contain and these revolved around factors that support its use and further action.

## Discussion

This project has been able to develop a definition of a RDAT that reached consensus after three rounds from a Delphi expert panel. Members of the panel were experts in knowledge mobilisation, policy makers, researchers and practitioners. Our consensus definition has therefore been informed by the tacit knowledge of individual participants made explicit through the consensus generating mechanism of a Delphi. The quality of the definition will be judged by others, and we recognise there is further work to be undertaken in order to test it empirically. We hope in time the definition will be refined, and the concepts contained within it further explored to understand more about the metaphorical know – do gap.

Delphi panels are helpful in reaching such consensus especially when the matter is complex and unclear as they act as virtual panel of expertise [18] but it is important to recognise there are limitations to the method and these are applicable to this study. The limitations centre around the ability to recruit a range of experts and maintain their involvement [19]. Our experts largely came from the knowledge producer, policy and practice community interested in implementation of research in practice. Future work needs to involve service users [24]. Considerations on context and facilitation in the use of RDATs should also be explored with a range of practitioners.

Another limitation was the focus and boundary of this research, which examined the research-practice gap in applied research with a focus on policy and practice. We recognise there are other areas were RDATs might be a useful concept for example, in basic science or in technologies and its link to applied research. This was not explored in this project.

We had drop out between rounds two and three of nearly one third, and this may be down to the extensive nature of round 2 which required consideration of many candidate RDAT. All were busy clinical-academics, and time pressures for competing the survey may be demanding in such schedules. There may have been disappointment in not pursuing agreement on types of tools in the third round, although this decision was based on the interim findings of round 2.

Further, the initial round workshop was shaped around two theoretic models in relation to knowledge mobilisation. This included Graham’s funnel mode [1, 25] in the Knowledge into action cycle, and the concept of RDATs as a boundary object. We recognise there are other models, and that the wider panel members may have been distracted by our conceptualisation in this way.

## Conclusion

Finally, there was a strong consensus that planning the research process, and the production of such research outputs should be undertaken in partnership with end users from writing grant applications onwards. This resonates with much of the knowledge mobilisation literature. For example, Kothari et al. promote a model of Integrated Knowledge Translation, where collaboration occurs from the beginning of the project onwards as end users have a unique knowledge of the context and the potential for implementation of the research [9]. Others support the theory of coproduction within the research process which views of the Delphi panel also reflected [26]. Coproduction demands that knowledge is generated within the context of use [27] and calls on a blurring of boundaries between this producers and users of research [26, 28] which also supports notions of co-production. There is also evidence that these more collaborative approaches are helpful in supporting impact in services [29], which may be helpful in evidencing how research has produced societal impacts, which is increasingly important for research communities and well as for practice. What this study adds is that an expert panel has endorsed this approach with respect to generating RDATs in addition to peer reviewed journals, and describes the characteristics of such outputs that have a potential for action and impact. It also provides a consensus about planning and protecting resources in the research process to do this.

An issue for debate arising from these findings is whether the academic community is prepared, or able to undertake such activity. Coproduction involves addressing power differences [30, 31], and involves skills such being able to communicate with different audiences including different disciplines and professions. It demands a balance of flexibility and creatively with maintaining standards of research rigor. It involves time, navigation around difficulties, and epistemological tolerance [14]. This requires further exploration, with considerations on how we develop these skills in the applied health research workforce.

The study results have relevance for both research funders and research producers. For funders, there are messages about providing resources in their calls for funding RDAT as a distinct element of research projects. Tender briefs and application processes could ask for evidenced of RDATs within the research design, and encouraging researchers to consider such outputs in dissemination strategies. For researchers there are recommendations about how to develop RDATs within the research process.

Our work has highlighted that achieving agreement on a typology of RDATs is more problematic. Our findings continue to iterate that ‘context is key’ in relation to whether a RDAT has a potential for impact and change. This is reinforced by the implementation science and knowledge mobilisation literature [32]. The findings also suggest that it is the end user who decides this. However, the expert panel have been able to identify key considerations that may support the implementation and use of RDATs. The reality is that there may well be no ‘magic bullet’, and that a number of approaches may be useful [29]. However these results suggest that funding the production of, and tracking the use of RDATs could be an important contribution to using research knowledge in practice.

## Additional files

Additional file 1: Delphi survey instrument used in round 2. (PDF 1478 kb)

Additional file 2: Delphi instrument used in round 3. (PDF 437 kb)

## Abbreviations

CCG: Clinical Commissioning Group
CLAHRC: Collaboration for Leadership in Health Research and Care
NHS: National Health Service
NICE: National Institute for Health and Care Excellence
NIHR: National Institute for Health Research
PPI: Patient and Public Involvement
RDAT: Research Derived Actionable Tool
YH: Yorkshire and Humber
