# Supplementary material for: What is a research derived actionable tool, and what factors should be considered in their development? A Delphi study
Source: BMC Health Serv Res. 2018 Sep 27;18:740. doi: 10.1186/s12913-018-3551-6 (PMC6161350; doi:10.1186/s12913-018-3551-6)
Supplement: Supplementary file 1 — Delphi survey instrument used in round 2. (PDF 1478 kb) [file 12913_2018_3551_MOESM1_ESM.pdf]

Determining the type of Actionable Tools that can be developed from NIHR research findings: A Delphi Study

Thank you for agreeing to be part of this Delphi Project. This is the first of two surveys, in this project, and we hope you will find it an opportunity to reflect on your experiences of getting research into practice.

The purpose of this study is twofold to:

1) clarify what is meant by the term 'actionable tool' as an output from projects
2) identifying and categorising such tools with the aim of developing a eRepository of such tools to support commissioning.

Our focus is outputs from NIHR funded research.

We are also interested in what needs to be considered in the production of an actionable research tool/output. This forms the final part of this Delphi study. We do recognise that the existence of an 'actionable' research output is only part of the story in terms of getting evidence into practice.

The Delphi panel, you have joined, consists of both academics and practitioners with an interest in and /or experience of getting evidence into practice.

INSTRUCTIONS
Generally, questions in this survey use a Likert scale where 1 = strongly agree and 5 = strongly disagree. There are also a number of open questions.

* Required

SECTION 1: TOWARDS A DEFINTION

This section begins with a definition that we developed during a workshop with a small group of colleagues (both academic and practitioner).

We consider an 'actionable tool' is a type of 'boundary object.' Fox (2011) describes a boundary object as 'a construct that has potential to improve the uptake transfer and innovation of research findings, technology and other intellectual property across the fields of social policy, organisation and management and commercial and public services.'

For us, a research derived 'actionable tool' has three elements:

KNOWLEDGE OBJECT: you can recognise or track knowledge contained in the tool which has been generated by the research.

MEDIUM FOR COMMUNICATION that is mindful of the 'target group' that the research findings might be pertinent to. Modes of communication are varied and could be electronic, paper, film etc.

PROMPT: they include overtly or inherently a PROMPT FOR ACTION that facilitates a decision to act or choose not to act.

The latter two elements may instigate different levels of (inter) activity offered by the tool.

Fox, N. J. (2011) Boundary Objects, Social Meanings and the Success of New Technologies. Sociology. 45 (1), 70–85. [online]. Available from: http://soc.sagepub.com/cgi/doi/10.1177/0038038510387196.

1. **To what extent do you agree with the above definition?** *
Mark only one oval.

	1	2	3	4	5	
strongly agree	⬭	⬭	⬭	⬭	⬭	strongly disagree

2. **Could you please explain your reasoning?**

3. **Regardless of the extent to which you agree with the above definition. How far do you agree that a research derived actionable tool will have distinct elements?** *
Mark only one oval.

	1	2	3	4	5	
strongly agree	⬭	⬭	⬭	⬭	⬭	strongly disagree

4. **Could you please explain your reasoning?**

5. **What other elements should be considered?**

6. **To what extent do you agree that an actionable tool should include each of the three elements contained within our definition?** *
1 = strongly agree, 5 = strongly disagree
Mark only one oval per row.

	1	2	3	4	5
Knowledge Object	⬭	⬭	⬭	⬭	⬭
Medium	⬭	⬭	⬭	⬭	⬭
Prompt	⬭	⬭	⬭	⬭	⬭

7. **Could you please explain your reasoning?**

8. **Our definition uses the term PROMPT and states that they include overtly or inherently a PROMPT for action that facilitates a decision to act or choose not to act. Can you give us examples of 'actionable tools' where you think the prompt is OVERT or APPARENT?**
   Please leave BLANK if no examples spring to mind

9. **Our definition uses the term PROMPT and states that they include overtly or inherently a PROMPT for action that facilitates a decision to act or choose not to act. Can you give us examples of 'actionable tools' where you think the prompt is INHERENT?**
   Please leave BLANK if no examples spring to mind

10. **Finally, in this section we are interested in other possible definitions of an actionable tool. For example, have we missed something really fundamental in your view. So, we would like you to give us a definition you are comfortable with. You may want to use our definition as a starting point.**

Page 2 of 5

SECTION 2: IDENTIFYING ACTIONABLE TOOLS

As part of our initial workshop, we used the above elements (knowledge object, medium and prompt) to decide whether items in generated list of research outputs could be considered to be 'actionable tools' according to our definition.

The list of research outputs was generated by workshop participants and a brief review of the

literature.

The next section asks you to look at examples (drawn from the literature) of research outputs and to decide the extent to which you agree they are 'actionable.' Further information about each example can be found in the links and/or reference.

DEFINTION REMINDER

A research derived 'actionable tool' has three elements:

KNOWLEDGE OBJECT: you can recognise or track knowledge contained in the tool which has been generated by the research.

MEDIUM FOR COMMUNICATION that is mindful of the 'target group' that the research findings might be pertinent to. Modes of communication are varied and could be electronic, paper, film etc.

PROMPT: they include overtly or inherently a PROMPT FOR ACTION that facilitates a decision to act or choose not to act.

The latter two elements may instigate different levels of (inter) activity offered by the tool.

11. **To what extent do you agree that a SERVICE SPECIFICATION is an 'actionable tool'?** *
Example: 'a model specification for children and adolescent community mental health services'
SOURCE: http://www.england.nhs.uk/wp-content/uploads/2015/01/mod-camhs-tier-2-3-spec.pdf
Mark only one oval.

	1	2	3	4	5	
strongly agree	◯	◯	◯	◯	◯	strongly disagree

12. **Could you please explain your reasoning?**

13. **To what extent do you agree that a SERVICE EVALUATION/RESEARCH TOOL is an 'actionable tool'?** *
For example, an evaluation toolkit SOURCE: http://www.apcrc.nhs.uk/evaluation/toolkit.htm
Mark only one oval.

	1	2	3	4	5	
strongly agree	◯	◯	◯	◯	◯	strongly disagree

14. **Could you please explain your reasoning?**

15. **To what extent do you agree that a WORKSHEET is an 'actionable tool'?** *

For example, an evidence based worksheet to support medicines management reviews for individual patients. SOURCE:http://psnc.org.uk/wp-content/uploads/2013/07/MUR-worksheet-following-2014-15-CPCF-changes.pdf

Mark only one oval.

	1	2	3	4	5	
strongly agree	⬭	⬭	⬭	⬭	⬭	strongly disagree

16. **Could you please explain your reasoning?**

17. **To what extent do you agree that a SIMULATION MODEL is an 'actionable tool'?** *

For example, a model on female obesity and fertility. SOURCE: Sabounchi, Nasim S., et al. "A novel system dynamics model of female obesity and fertility." American journal of public health 104.7 (2014): 1240-1246.

Mark only one oval.

	1	2	3	4	5	
strongly agree	⬭	⬭	⬭	⬭	⬭	strongly disagree

18. **Could you please explain your reasoning?**

19. **To what extent do you agree that a CLINICAL DECISION AID is an 'actionable tool'?** *

For example, a rule-based expert system for a heart failure mobile phone-based telemonitoring system. SOURCE: Seto, Emily, et al. "Developing healthcare rule-based expert systems: case study of a heart failure telemonitoring system." International journal of medical informatics 81.8 (2012): 556-565.

Mark only one oval.

	1	2	3	4	5	
strongly agree	⬭	⬭	⬭	⬭	⬭	strongly disagree

20. **Could you please explain your reasoning?**

21. **To what extent do you agree that an AUDIT TOOL is an 'actionable tool'?** *
For example, an audit tool to support nutritional quality improvement. SOURCE: Heyland, Daren K., et al. "Driving perioperative nutrition quality improvement processes forward!." Journal of Parenteral and Enteral Nutrition 37.5 suppl (2013): 83S-98S.
Mark only one oval.

	1	2	3	4	5	
strongly agree	⬭	⬭	⬭	⬭	⬭	strongly disagree

22. **Could you please explain your reasoning?**

Page 3 of 5

23. **To what extent do you agree that an EXECUTIVE SUMMARY is an 'actionable tool'** *
For example, an executive summary of research on improving patient flow in an emergency department. SOURCE:http://www.ahrq.gov/research/findings/final-reports/ptflow/executive-summary.html
Mark only one oval.

	1	2	3	4	5	
strongly agree	⬭	⬭	⬭	⬭	⬭	strongly disagree

24. **Could you please explain your reasoning?**

25. **To what extent do you agree that a PATIENT DECISION AID is an 'actionable tool'? ***

For example a 'comprehensive evidence-based multiple sclerosis booklet about immunotherapy options and an interactive worksheet. SOURCE: Köpke, Sascha, et al. "Information provision for people with multiple sclerosis." The Cochrane Library (2014).
Mark only one oval.

	1	2	3	4	5	
strongly agree	◯	◯	◯	◯	◯	strongly disagree

26. **Could you please explain your reasoning?**

____
____
____
____
____

27. **To what extent do you agree that an ALGORITHM FOR CLINICAL DECISION MAKING is an 'actionable tool'? ***

For example,'electronic clinical decision support in the form of an algorithm SOURCE: Mitchell, Marc, et al. "Improving care-improving access: the use of electronic decision support with AIDS patients in South Africa." International Journal of Healthcare Technology and Management 10.3 (2009): 156-168.
Mark only one oval.

	1	2	3	4	5	
strongly agree	◯	◯	◯	◯	◯	strongly disagree

28. **Could you please explain your reasoning?**

____
____
____
____
____

29. **To what extent do you agree that an RISK ASSESSMENT TOOL is an 'actionable tool'? ***

For example, a risk assessment tool to support cancer diagnosis. SOURCE: Rubin, Greg, et al. "Research into practice: prompt diagnosis of cancer in primary care." British Journal of General Practice 64.625 (2014): 428-430.
Mark only one oval.

	1	2	3	4	5	
strongly agree	◯	◯	◯	◯	◯	strongly disagree

30. **Could you please explain your reasoning?**

31. **To what extent do you agree that a BALANCED SCORECARD is an 'actionable tool'?** *

For example, a balanced scorecard used to support performance SOURCE: Kollberg, Beata, and Mattias Elg. "The practice of the Balanced Scorecard in health care services." International Journal of Productivity and Performance Management 60.5 (2011): 427-445.

Mark only one oval.

	1	2	3	4	5	
strongly agree	◯	◯	◯	◯	◯	strongly disagree

32. **Could you please explain your reasoning?**

33. **To what extent do you agree that a TEACHING AND LEARNING PACK is an 'actionable tool'?** *

For example, an e-learning package for professionals. SOURCE: Murphy, Jane, et al. "Translating research into practice: Evaluation of an e-learning resource for health care professionals to provide nutrition advice and support for cancer survivors." Nurse education today 35.1 (2015): 271-276.

Mark only one oval.

	1	2	3	4	5	
strongly agree	◯	◯	◯	◯	◯	strongly disagree

34. **Could you please explain your reasoning?**

35. **To what extent do you agree that a LOCAL PROTOCOL is an 'actionable tool'?** *

For example, local guidance/protocol on antibiotic use.
SOURCE:http://www.bassetlawccg.nhs.uk/publication/6999-doncaster-and-bassetlaw-antibiotic-guideline-2015#

Mark only one oval.

	1	2	3	4	5	
strongly agree	⬭	⬭	⬭	⬭	⬭	strongly disagree

36. **Could you please explain your reasoning?**

37. **To what extent do you agree that a NATIONAL PROTOCOL OR GUIDELINE is an 'actionable tool'?** *

For example, NICE guidance on antimicrobial stewardship.
SOURCE:https://www.nice.org.uk/guidance/ng15

Mark only one oval.

	1	2	3	4	5	
strongly agree	⬭	⬭	⬭	⬭	⬭	strongly disagree

38. **Could you please explain your reasoning?**

39. **To what extent do you agree that SOCIAL MARKETING MATERIALS are 'actionable tools'?** *

For example, a pen-portrait to depict findings. SOURCE: http://kwillt.org/index.php/pen-portraits

Mark only one oval.

	1	2	3	4	5	
strongly agree	⬭	⬭	⬭	⬭	⬭	strongly disagree

40. **Could you please explain your reasoning?**

41. **To what extent do you agree that a FILM is an 'actionable tool'?** *
For example, a film as part of an e-learning package SOURCE:Henry, R., et al. "The development and evaluation of a holistic needs assessment and care planning learning package targeted at cancer nurses in the UK." ecancermedicalscience 8 (2014).
Mark only one oval.

	1	2	3	4	5	
strongly agree						strongly disagree

42. **Could you please explain your reasoning?**

43. **To what extent do you agree that a PATIENT REPORTED OUTCOME MEASURE (PROM) is an 'actionable tool'?** *
For example, a PROM on quality of life for children. SOURCE:
https://www.shef.ac.uk/polopoly_fs/1.44111!/file/Health-Questionnaire-final-watermarked.pdf
Mark only one oval.

	1	2	3	4	5	
strongly agree						strongly disagree

44. **Could you please explain your reasoning?**

45. **To what extent do you agree that a PATIENT REPORTED EXPERIENCE MEASURE (PREM) is an 'actionable tool'?** *

For example, a PREM for urgent and emergency care. SOURCE: http://www.rcpch.ac.uk/final-urgent-and-emergency-care-prem-tools

Mark only one oval.

	1	2	3	4	5	
strongly agree	◯	◯	◯	◯	◯	strongly disagree

46. **Could you please explain your reasoning?**

47. **We know that our examples are not an exhaustive list. Do you consider any other outputs from research as 'actionable tools'?**

Please list any examples and if possible include links/references. We are particularly looking for examples from NIHR funded research.

Page 4 of 5

SECTION 3: STEPS REQUIRED TO DEVELOP ACTIONABLE TOOLS

We recognise that research derived outputs are not automatically actionable and that getting knowledge into action is complex. We also recognise developing actionable tools as a research output is not the whole of the story.

48. **What ingredients do you think help turn a 'potentially actionable research communication' into an 'actionable tool'**

For example, what would need to be in place to make for example an 'executive summary' an 'actionable tool' or boundary object.

49. **The workshop suggested that 'actionable tools' are improved by co-producing these with end users. What do you think is meant by this?**

50. **How far do you agree that the following statements would help the development of 'actionable tools' as part of a research grant submission?** *

1 = strongly agree and 5 = strongly disagree
Mark only one oval per row.

	1	2	3	4	5
The research team consider their audience (s) at grant application					
The research team involves their audience (s) in the research design					
The grant includes resources to undertake 'actionable tool' development					
The production of 'actionable' research outputs is a condition of the research grant					

51. **What skills do you think research teams need to develop 'actionable tools'?**

52. **What resources do you think research teams need to develop 'actionable tools'?**

53. **Are there any governance issues we should consider?**

54. **Have we missed any important issues?** *
Check all that apply.

- [ ] yes
- [ ] no

55. **If yes, please could you explain?**

____
____
____
____
____

SECTION 4: INFORMATION ABOUT YOU

To aid our analysis it would be helpful if we had some additional information about you.

56. **What is your primary role?** *
Check all that apply.

- [ ] academic
- [ ] practitioner
- [ ] academic-practitioner
- [ ] commissioner
- [ ] policy maker
- [ ] boundary spanner
- [ ] Other: ________________________________

57. **Finally, please insert your email address** *
this is simply for administrative purposes

____

Powered by
Google Forms
