# Supplementary material for: What is a research derived actionable tool, and what factors should be considered in their development? A Delphi study
Source: BMC Health Serv Res. 2018 Sep 27;18:740. doi: 10.1186/s12913-018-3551-6 (PMC6161350; doi:10.1186/s12913-018-3551-6)
Supplement: Supplementary file 2 — Delphi instrument used in round 3. (PDF 437 kb) [file 12913_2018_3551_MOESM2_ESM.pdf]

Determining the type of Actionable Tools that can be developed from NIHR research findings: A Delphi Study

Welcome to the second Delphi survey which is considerably shorter than the first round. Once again, we would like to thank you for your considered responses which encompassed both practical and philosophical insight. We have used this insight to develop this next round. A brief summary of our findings can be found in the covering email.

This study is concerned with identifying and depositing products from NIHR funded research into an e-repository with aim of closing the Knowledge-Action gap.

This second round then focuses on refining our definition as it was considered to be too abstract for the intended audience. We had consensus that an actionable tool would have the elements we identified but our analysis of your contributions also identified that there may be other considerations. We have asked additional questions about this.

As a reminder, the Delphi panel, you have joined, consists of both academics and practitioners with an interest in and /or experience of getting evidence into practice.

INSTRUCTIONS
Generally, questions in this survey use a Likert scale where 1 = strongly agree and 5 = strongly disagree. There are also a number of open questions.

* Required

Section 1: A clearer definition

We were near consensus with the definition used in the last round but were advised by the panel, that the language we used, was too academic and may be off putting to our audience. We have, therefore, developed a new definition drawing on the numerous suggestions of the Delphi Panel. We have retained central concepts around elements as we gained clear consensus on these issues.

OUR NEW DEFINITION

A research derived actionable tool is a product informed by research study findings that is intended to change the way of thinking, promote decision making or instigate action around an issue. Actionable tools are characterised by:

RESEARCH KNOWLEDGE : you can recognise the research findings within the tool.

MEANS of COMMUNICATION: the approach to communicating the findings shows consideration of the target audience, and how they prefer to receive information.

PROMPT: the tool invites the target audience to reflect on the research findings and triggers appropriate action within the local setting.

1. **To what extent do you agree with the above definition? ***
1 = strongly agree, 5 = strongly disagree
Mark only one oval.

	1	2	3	4	5	
strongly agree	◯	◯	◯	◯	◯	strongly disagree

2. **Please explain your reasons**

Section 2: other considerations

Your responses have highlighted that there are other factors which may be worth consideration in the shaping, content and dissemination of such tools.

We have organised these considerations into two themes; considerations which are about the NIHR research study itself and considerations around the research derived product.

We ask you to consider how important these considerations are to potential end users of the tool.

The research itself

3. **To what extent do you agree that the following considerations are important to end users of an actionable tool?**

Mark only one oval per row.

	strongly agree	agree	neither agree nor disagree	disagree	strongly disagree
the research funding body					
the research team that has undertaken the work					
the institution(s) that has/have undertaken the work					
the setting/ context of the research study					
the methods used					
explanation as to what the study adds to the evidence base					
explanation of the reasoning underpinning the chosen study methods					

4. **Please explain your reasons**

The tool itself

5. **To what extent do you agree that the following considerations are important to end users of an actionable tool?**
Mark only one oval per row.

	strongly agree	agree	neither agree nor disagree	disagree	strongly disagree
information about who needs to take action	⬭	⬭	⬭	⬭	⬭
ability to tailor to the local context	⬭	⬭	⬭	⬭	⬭
information about outcomes measures that might be used as part of putting the tool into practice	⬭	⬭	⬭	⬭	⬭
information about the support available to implement the tool	⬭	⬭	⬭	⬭	⬭
testimonies from other users	⬭	⬭	⬭	⬭	⬭
self assessment guidance for using the tool	⬭	⬭	⬭	⬭	⬭
includes evaluation tools	⬭	⬭	⬭	⬭	⬭
prioritises the actions	⬭	⬭	⬭	⬭	⬭
timescales for implementation	⬭	⬭	⬭	⬭	⬭

6. **Please explain your reasons**

____

____

____

____

____

7. **Finally, we intend to publish our findings and need to know if you are willing to be acknowledged in these publications?**
Mark only one oval per row.

	Yes	No	Unsure (please contact me to discuss)
Report to NHS England (our funders)	⬭	⬭	⬭
Academic journals	⬭	⬭	⬭
Academic conferences	⬭	⬭	⬭
Blogs	⬭	⬭	⬭

Thank you once again for your help with this work.

Powered by
Google Forms
